# Functional and Homeostatic Impact of Age-Related Changes in Lymph Node Stroma

**DOI:** 10.3389/fimmu.2017.00706

**Published:** 2017-06-14

**Authors:** Heather L. Thompson, Megan J. Smithey, Charles D. Surh, Janko Nikolich-Žugich

**Affiliations:** ^1^Department of Immunobiology, The Arizona Center on Aging, University of Arizona College of Medicine, Tucson, AZ, United States; ^2^Academy of Immunology and Microbiology, Institute of Basic Science, Pohang, South Korea; ^3^Department of Integrative Biosciences and Biotechnology, Pohang University of Science and Technology, Pohang, South Korea; ^4^Division of Developmental Immunology, La Jolla Institute of Allergy and Immunology, La Jolla, CA, United States

**Keywords:** aging, immunity, lymph nodes, fibroblastic reticular cells, lymphatic endothelial cells, naïve T cells

## Abstract

Adults over 65 years of age are more vulnerable to infectious disease and show poor responses to vaccination relative to those under 50. A complex set of age-related changes in the immune system is believed to be largely responsible for these defects. These changes, collectively termed immune senescence, encompass alterations in both the innate and adaptive immune systems, in the microenvironments where immune cells develop or reside, and in soluble factors that guide immune homeostasis and function. While age-related changes in primary lymphoid organs (bone marrow, and, in particular, the thymus, which involutes in the first third of life) have been long appreciated, changes affecting aging secondary lymphoid organs, and, in particular, aging lymph nodes (LNs) have been less well characterized. Over the last 20 years, LN stromal cells have emerged as key players in maintaining LN morphology and immune homeostasis, as well as in coordinating immune responses to pathogens. Here, we review recent progress in understanding the contributions of LN stromal cells to immune senescence. We discuss approaches to understand the mechanisms behind the decline in LN stromal cells and conclude by considering potential strategies to rejuvenate aging LN stroma to improve immune homeostasis, immune responses, and vaccine efficacy in the elderly.

## Introductory Remarks

Older adults exhibit a greater susceptibility to infection and reduced responses to vaccination relative to young adults, and infectious diseases remain among the leading causes of morbidity and mortality in the elderly (>65 years of age) (1). While multiple changes occur in the organism with aging, immune senescence is believed to be the key culprit for this susceptibility. Immune senescence affects both the innate and adaptive branches of the immune system, as well as the stromal microenvironments that affect T cell development and homeostasis (2–4). It has been well established that the thymus begins involution relatively early in life, becoming progressively smaller, more disorganized, and functionally inferior, with reduced naïve T cell output (5). The changes in output of naïve T cells from the aging thymus have long been associated with the numerical decline in naïve T cells in the periphery of aged animals, while memory T cells accumulate proportionally (4). However, memory cells do not increase in absolute numbers with aging unless persistent infection with cytomegalovirus is also present (6). Substantial research has dissected the changes that occur to both T cell development with age (5) and to peripheral T cell homeostasis and function (2). However, less attention has been paid to the aging stromal environment that is expected to maintain these lymphocytes throughout the lifespan. Here, we discuss the series of changes that affect the aging lymph node (LN) architecture and function as a critical factor contributing to poor age-associated immune responses and propose new therapeutic targets to rejuvenate the aging immune system.

## Function and Organization of LN Stroma

The primary function of the LN is to coordinate immune responses to antigens trafficking from peripheral tissues. The non-hematopoietic stromal cell subsets provide the architecture and scaffolding necessary to guide cellular trafficking and compartmentalization, facilitate antigen presentation to circulating naïve T and B cells and thereby promote immune surveillance against infection. In addition, LN stromal cells are responsible for the production and presentation of chemokines that coordinate this trafficking of lymphocytes into and throughout the LN (7, 8). LN stromal cells also provide a crucial microenvironment for immune homeostasis and lymphocyte maintenance *via* presentation of pro-survival cytokines such as IL-7 and IL-15 to T cells (7, 8), and CXCL13 and B-cell activating factor of the TNF family (BAFF) to B cells (9).

### Phenotypic Characteristics of LN Stromal Cells

**Figure 1:**
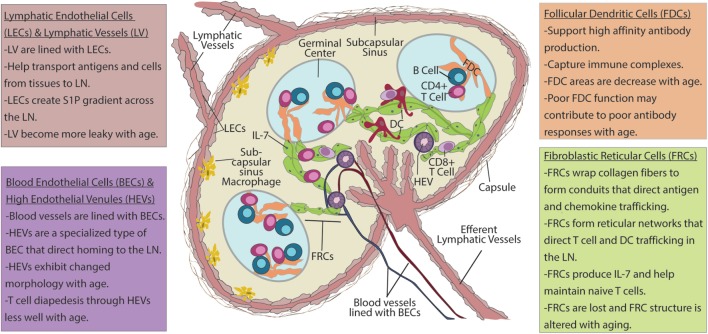
Lymph node (LN) stroma elements and their changes with aging. *Upper left box*: lymphatic vessels (LVs) are lined with lymphatic endothelial cells (LECs). These vessels transport antigens and cells from peripheral tissues to draining LNs. LECs also produce sphingosine-1-phosphate (S1P) that forms a chemotactic gradient for migration of T cells into efferent lymphatics. Migratory dendritic cells (DCs) enter LN *via* LVs and into subcapsulary sinuses (SCSs) before entering the LN parenchyma. As antigens drain into the SCS, which are also lined with LECs, SCS macrophages pick up antigen and transfer it to follicular dendritic cells (FDCs). *Upper right box*: FDCs present immune complexes to B cells to enhance high-affinity antibody formation. *Lower left box*: blood endothelial cells (BECs) line blood vessels that transport blood borne cells into LN. High endothelial venules (HEVs) are specialized BECs with cuboidal morphology, T cell diapedesis across HEVs to enter the LN parenchyma. *Lower right box*: after entering the LNs naïve T cells from the blood stream crawl along fibroblastic reticular cells (FRCs) that form the reticular network in search of DCs bearing cognate peptide–MHC and costimulation to become activated. FRCs also have critical roles in the maintenance of naïve T cells through the production chemokines and IL-7. *Age-related changes*: with age, LVs become leaky and less capable of facilitating movement between of cells and antigens between the peripheral tissues and the LN to coordinate immune response. HEVs have altered morphology with age, and T cells have difficulty moving across HEVs with increased age. FRCs exhibit numerical reduction as well as disorganization of reticular networks with aging. This is likely to impair naïve T cell homeostasis, as well as movement of T cells within the LN and may impact the ability of aged LN to generate productive T cell responses. FDC areas are also reduced with age. Changes to FDCs may contribute to poor affinity of antibody responses that are observed with increased age.

The stromal cells of the LN are a numerically small, CD45^−^TER119^−^ population derived from endothelial and mesenchymal progenitors, relative to hematopoietic-derived CD45^+^ or TER119^+^ cells, which make up the vast majority (>98%) of LN cells (10) (Ter119 marks red blood cells). Within the stromal fraction, cell surface expression of podoplanin (PDPN, also known as gp38), CD31 (PECAM-1), and CD35/CD21 (complement receptor 1 and 2) distinguish five major, functionally important subsets: fibroblastic reticular cells (FRCs; gp38^+^CD31^−^CD35/CD21^−^), lymphatic endothelial cells (LECs; gp38^+^CD31^+^CD35/CD21^−^), blood endothelial cells (BECs; gp38^−^CD31^+^CD35/CD21^−^), follicular dendritic cells (FDCs; gp38^±^CD31^−^CD35/CD21^+^), and double/triple negative (DN) cells (gp38^−^CD31^−^CD35/CD21^−^) (11, 12) (Figure 1; Table 1).

**Table 1:** Age-related changes to lymph node (LN) stromal cell populations.

Cell type	Markers	Known functions	Changes with age
Fibroblastic reticular cells (FRCs)	gp38^+^, CD31^−^, CD35/CD21, CD45^−^, Ter119^−^ ER-TR7^+^ in histology	Help form conduits and reticular network Regulate naïve T homeostasis Regulate naïve T cell movement Secrete CCL19, CCL21, and CXCL12 IL-7 presentation	Becklund et al. found that FRCs are decreased in aging LN in homeostasis (13), while Turner and Mabbott found that FRC numbers are unchanged (14) FRC structure altered (13)

Follicular dendritic cells (FDCs)	CD35/CD21^+^, gp38^+/−^, CD31^−^, CD45^−^, Ter119^−^	Make reticular network for B cells FDC secrete CXCL13 Support production of high-affinity antibodies Capture immune complex	FDC area decreased in aged mice (14) Less CXCL13 produced in aged mice (protein) (14) More CXCL13 expressed in aged mice by qPCR (13) Less CXCL13 produced in response to infection in aged mice (15)

Double negative stromal cells (DN)	gp38^−^, CD31^−^, CD35^−^, CD45^−^, Ter119^−^	Thought to be FRC like pericytes Function of these cells is mostly unknown	Decreased in number in aged mice (14)

Blood endothelial cells (BECs)	gp38^−^, CD31^+^, CD35^−^, CD45^−^, Ter119^−^	BECs construct cortical blood vessels and capillaries, including high endothelial venules (HEVs)	Unchanged between old and adult mice (14)

HEVs	These are a type of BEC PNAd^+^ in histology	Main route of entry for lymphocytes HEVs have cuboidal morphology	Impaired T cell diapedesis at aged HEV (13, 15) HEVs reported as more dense and compressed in aged LN (13)

Lymphatic endothelial cells (LECs)	gp38^+^, CD31^+^, CD35^−^, CD45^−^, Ter119^−^ LYVE-1^+^ in histology	Transport antigens and lymph from peripheral tissues to LN. Connection between LN Help create sphingosine-1-phosphate gradient across LN	No change in LECs (14).

Lymphatic vessels (LVs)	LYVE-1^+^	Transport antigens, immune cells, and lymph from peripheral tissues to LN	LV showed a 20% decrease in contraction amplitude and a 70% decrease in contraction frequency (16) LV leakiness and impaired pathogen clearance in aged mice between footpad and popliteal LN (16)

### Functional Characteristics of LN Stromal Subsets

The endothelial derived LECs and BECs help mediate transport of both circulating cells and tissue-derived antigens into and out of the LNs. Entry into the lymphatics from the tissues occurs through lymphatic collectors and vessels lined with LECs (17). LECs also line sinuses in the LNs delivering antigen from the tissues and providing a route for cells to travel to the next LN (18). In general, BECs line blood vessels. A specialized BEC subset, called HEVs facilitates entry of circulating lymphocytes into the LN *via* a multistep adhesion and extravasation process utilizing chemokines, selectins, addressin and integrins (18).

Mesenchymal cells create the reticular network within the LN and are critical for the maintenance of its architecture; FRCs, FDCs, and DN stromal cells partake in this task. FRCs are a specialized type of reticular fibroblast that create a large proportion of the stromal network within the LN (19). FRCs ensheath bundles of collagen fibers to create conduits for the transport of small molecules, including antigens/antigen complexes and provide a transport system that guides DC and T cell movement (20). FDCs are also specialized reticular fibroblasts (9) that secrete CXCL13, guiding B cells, and follicular helper T cells into the germinal center (GC) to facilitate high-affinity antibody production (21). While the function of DN/TN cells is largely unknown, gene profiling studies suggest that some of these cells may be mesenchymal progenitors, consistent with their positioning as pericytes (20, 22). Pericytes within the double negative fraction may also help regulate blood vessel integrity, as well as permeability within the LN (22).

### Hematopoietic Cells Facilitate LN Stroma Maintenance

Lymph node stromal cells have close bidirectional relationships with hematopoietic cells, each contributing to the homeostasis of the other (23). Innate lymphoid cells (ILC) are a broad category of cells that develop from common lymphocyte progenitors but do not have rearranged antigen receptors (24). ILC include lymphoid tissue inducers (LTi), which are a sub-group of ILC group 3 cells (25). During LN development, LTi are an important source of lymphotoxin beta (LTβ), which combines with lymphotoxin alpha to make the heterotrimer LTα_1_β_2_ (25, 26). This heterotrimer can signal mesenchymal stem cells through the LTβ receptor (LTβR) to differentiate into lymphoid tissue organizers, which are critical in inducing proper development and architecture formation of other stromal cells, particularly FRC. Although LTi were originally recognized for their role in LN developmental, they are present in the adult LN and appear to also mediate adult tissue regeneration (24). LTi help induce regeneration of FRC networks in the spleen and LN following lymphocytic choriomeningitis virus infection (27). It should be noted that while LTi are an important source of LT, other lymphocytes including T, B, and NK cells also secrete LT and contribute to LT availability in the LN (28). Therefore, it is possible, and indeed likely, that naïve T and/or B cells contribute to the health and maintenance of FRC and other stromal cells, which, in turn, provide trophic factors for naïve lymphocyte survival and maintenance.

Other signals from hematopoietic populations in the LN influence the structure, function, repair, and regeneration of LN stroma. C-type lectin receptor 2 (CLEC-2) is expressed by megakaryocytes, platelets, neutrophils, DCs, and NK cells (12, 29). CLEC-2 serves as a ligand for PDPN expressed on stromal cells and triggers the relaxation of FRC networks (30), which in turn impacts how many antigen specific T cells can be recruited into the LN to respond (31). FRC lines isolated from LN are dependent on lymphocytes for production of ER-TR7 [which identifies the extracellular matrix (ECM) produced by FRC, but the antigen has not been identified]; reticular networks fail to form in the absence of this interaction *in vitro* (7, 23). Therefore, a picture is emerging of intense cross talk between hematopoietic and stromal cells, critical to the homeostasis and function of both compartments in the LN, although many mechanistic details still remain to be defined.

## Functional Consequences of Age-Related Changes to LN Stromal Cells

### Stromal Cells in Aged LN

While the contribution of LN stromal cells to both immune homeostasis and function is evident, age-related changes affecting stromal cells have been under investigated (32). Therefore, age-related dysfunction and/or disorganization of LN stromal cells may be an underappreciated contributor to immune senescence. Several groups have described chronic and progressive changes that occur in LN with age (33–35). In general, with aging, LN in both mouse and man become smaller and less cellular (33). Similar to thymic involution, histological studies of LN highlight that the organization is less distinct (especially between T and B cell areas) (13, 14), with an accumulation of adipocytes (33) and signs of fibrosis (34). Similar disorganization between T and B cell areas occurs in the aging spleen (36). It should be noted that not all LNs undergo the same age-related changes; skin-draining LNs are more affected than mucosal LN (33). Below, we discuss key defects in aging LN stroma that have been identified to date.

## Transport in and out of LN

### LVs and LECs

Afferent LV function as conduits for trafficking of both antigens and immune cells. DCs that have captured antigen in tissues move *via* LVs from peripheral tissues into draining LN (16). Imaging studies demonstrated that aged mice show a diminished capacity to transport bacteria (*Cryptococcus neoformans, Mycobacterium smegmatis*, and *Staphylococcus aureus*) from peripheral tissues into the draining LN, as seen by bacteria leaking out of lymphatics and into the surrounding tissue (16). This was due to both increased LV permeability (an LEC defect) and reduced contractility of the musculature that surrounds the LVs (16). Using paraquat to induce oxidative stress to LECs in a transwell system, the same study found increased LEC permeability to FITC–dextran (16). The authors proposed that the impaired *in vivo* bacterial transport was caused by increased oxidative stress to LECs (16).

Within the LN, the lymph enters through afferent lymphatics into subcapsulary sinus (SCS) lined with LECs and SCS macrophages (SCSM) (18). LECs provide routes in and out of the LN while the SCSM trap pathogens, antigen, and immune complex as they come into the LN (14, 37). Thus, the SCSM network, positioned at the entry of afferent lymphatics, acts to reduce pathogen dissemination and to increase the chance that antigen-presenting cells will come into contact with the rare T cell that might recognize them (37). SCSM additionally transfer incoming immune complexes to non-cognate B cells, which then transfer the complexes to FDCs (38). FDCs shuttle these antigens into non-degradative endosomal compartments, allowing long-term retention and presentation of the antigens (38). Turner and Mabbott showed that aged mice exhibit a significant increase in SCSM as a fraction of hematopoietic cells (14). Despite this increase in SCSM, the FDCs in aged mice fail to retain immune complexes (14). Further research is needed to address whether in old mice this increased SCSM population fails to efficiently capture/handoff antigen to FDCs, or whether these antigens are being shuttled into degradative endosomes, rather than the usual non-degradative endosomes that allows immune complexes to be retained by FDCs.

### BECs and HEVs

Circulating naïve B and T cells enter the LN through HEVs, which are a specialized subtype of BEC (18). HEVs have a cuboidal shape and a polarized expression of adhesion molecules so that circulating cells in the blood can anchor to the HEVs and extravasate into the LN (39). BECs (including HEVs) appear to be unchanged numerically in aged mice (14); however, aged HEVs appear to have a more dense and compressed morphology (13). There is some evidence that aged BECs show changes similar to that of the aging vascular system, including increased permeability, inflammation, and number of senescent endothelial cells (32, 40). Moreover, aged HEVs may poorly facilitate lymphocyte entry into the aged LN, based on experiments showing pronounced defects in recruiting adoptively transferred adult naïve T cells into old LN (13, 15).

## Cells of the Reticular Network

The reticular network provides the structure and architecture of the LN (23). The reticular network is composed of reticular fibers, ECM, and mesenchymal lineage cells such as FRCs and FDCs. Collectively, the reticular network creates specific microanatomical sites within the LNs that support and coordinate immune cells through the production of cytokines and chemokines (41).

### Fibroblastic Reticular Cells

Fibroblastic reticular cells are a category of cells representing at least five different populations (10). FRCs are uniquely important in both organizing the T cell zone within the LN and in maintaining naïve T cell viability and function. The conduits formed by FRCs extend across the T cell zone from the SCS to the HEVs and construct the reticular network of the LN (37). FRCs are specialized myofibroblast (10) that, like other myofibroblasts, express α-smooth muscle actin (7). Unlike other myofibroblasts, FRCs ensheath ECM-like collagen bundles, whereas fibroblasts in connective tissues are embedded within the ECM (23). Also, FRCs have higher expression of genes involved in cytokine signaling, as well as genes involved in antigen presentation pathways (42). FRCs can directly present antigens to promote either T cell activation or T cell peripheral tolerance (37). FRC expression of chemokines CCL21 and CCL19 controls T cell motility. It has been proposed that CCL21 interacts in a unique manner with glycosaminoglycans on FRCs to facilitate T cell movements (37). Specifically, CCL21 has a 32 amino acid long C-terminal tail containing 12 basic amino acid residues. This allows it to bind to glycosaminoglycans and other molecules like PDPN, a proteoglycan expressed by LECs and FRCs (43).

Naïve T cells decline in number with age (2). This has been primarily attributed to age-related thymic involution and the consequent decline in new naïve T cells produced. However, naïve T cells can have a long lifespan if provided the appropriate survival signals (7, 44). Link et al. demonstrated that FRCs play a key role in naïve T cell survival *via* production and presentation of IL-7 and CCL19 (7). Genetic knockout or antibody-mediated depletion of IL-7 results in a gradual loss of peripheral naïve T cells, whereas IL-7 transgenic mice exhibit a larger naïve T cell pool (45). Bajénoff et al. used intravital microscopy to show that T cells enter the LN *via* HEVs, then use FRCs to crawl to the LN parenchyma (19). When FRCs are depleted [e.g., in CCL19-diphteria-toxin (CCL19-DTR) mice], the total cellularity of the LN declines, with a significant loss of T cells beginning 24 h after FRC depletion (46).

Becklund et al. extended these findings testing whether LN in old mice can support adult T cell homeostasis. Both naïve TCR- transgenic and polyclonal populations from adult donors failed to survive, and proliferated less in old LN compared to adult LN after transfer (13). Further, old LN exhibited reduced numbers of FRCs, and their reticular network appeared less reticular and more condensed than in adults (13). Despite possessing normal levels of IL-7 mRNA in LN and IL-7 protein in circulation in old individuals, naïve T cells parked in old hosts exhibited lower levels of phosphorylated signal transducer and activator of transcription 5, a signaling molecule downstream of IL-7R (13). The discrepancy between the levels of IL-7 and the homeostatic signals received by naïve T cells suggested that IL-7 presentation by FRCs is altered in aged microenvironments. Mechanistic understanding of the naïve T cell maintenance programs across aging and across species (47) will be exceptionally important to immune rejuvenation strategies.

In addition to these problems of homeostatic maintenance, aged FRCs likely also contribute to the compromised immune responses to infection. An adult LN can expand up to 10-fold during an infection (48). Old LNs expand modestly, but never reach the cellularity seen during an adult immune response (15, 49). During infection, chemokines and cytokines are transported through FRC networks to HEVs where they are transcytosed and displayed on the luminal side of HEVs to recruit naïve T cells (50). Antigen-bearing DCs crawl first through LV into LN through afferent lymphatics, then along FRCs in search of their cognate T cell. Loss of LN organization and boundaries between B and T cell zones make the host more susceptible to infection (50). Depletion of FRC in adult CCR19-DTR mice resulted in reduced responses to replication incompetent influenza A or to a coronavirus-based vector, likely due to the reduction in chemokines reaching HEVs to recruit lymphocytes into the LN, and/or a reduction in coordination of immune cells within the LN (46). Somewhat unexpectedly, FRC depletion also had a profound negative impact on both B cell homeostasis (reduced B cell follicle size and disrupted T/B boundary), and decreased T-dependent and T-independent antibody responses.

Fibroblastic reticular cells are also a key source of collagen in the LN. Appropriate thickness and abundance of collagen, which is essential as part of the ECM, is an important physiological parameter of organ architecture and function (51). Increased thickness and abundance of collagen fibers, termed fibrosis, is a frequent change to many organs during aging and is associated with impaired function (52). Wound-healing cytokines, dominantly TGFβ and type 2 cytokines like IL-13 (53), induce fibrosis in LN and other organs and are also known to be increased with aging (52, 54). This process is believed to operate *via* FRC in some pathogenic conditions (55, 56) and is likely to also occur in the same manner during aging.

### Follicular Dendritic Cells

Follicular dendritic cells are specialized cells of mesenchymal origin (10, 57), named for their long cytoplasmic “dendritic” processes and are unrelated to classical hematopoietic DC (58, 59). The role of FDCs during homeostasis is less clear, but during infection FDCs support B cell movement and proper localization to GCs by producing CXCL13, as well as B-cell activating factor of the TNF family (BAFF) and a proliferation-inducing ligand (50). FDCs additionally help generate high-affinity antibody responses (10) by allowing prolonged antigen presentation to B cells undergoing somatic hypermutation (38).

Defects in FRCs and FDCs are a potential factor underlying poor humoral immunity in the elderly. Antibody responses are of lower affinity and impaired function compared to young adults (60). For example, during chikungunya virus infection, high antibody titers are found in old mice, but they show poor neutralizing function compared to adults (49). Further, aging results in fewer B cells within the LN (49), and B cell localization is less defined (13, 14). GC formation is reduced in the LN of aged mice infected with West Nile virus relative to adult controls (15). GC size has also been reported to decline with age in humans (33). FDCs are responsible for coordinating these events but have a decreased area in LN of aged mice compared to their adult counterparts (14), and less CXCL13 protein is produced in response to infection and in homeostasis (14, 15). Turner and Mabbott also found that immune complexes were retained less by aged FDCs (14). This loss of antigen may suggest that a degradative endocytic pathway is being used by aged FDCs, although this has not been directly demonstrated. These observations are consistent with the idea that age-associated impairments in FDCs (14) are a contributing factor to poor antigen retention, impaired GC formation, and decline in the production of high-affinity, functionally neutralizing antibodies.

## Mechanisms behind LN Involution and Rejuvenation

### Understanding the Mechanisms behind LN Involution

As mentioned above, the mechanisms driving LN changes with aging remain incompletely understood. Heterochronic parabiosis, the surgical joining of two organisms of different ages (adult and old) (3, 61), can be a powerful tool to discern cell-intrinsic vs. to cell-extrinsic (environmental) defects that occur with age (3). Both pro-geronic (62) and anti-geronic (63) factors have been identified using this technique. Using heterochronic parabiosis, we found a surprisingly marked loss of naïve T cell maintenance in the LN of the adult parabiont, with numbers reduced to that in the old parabiont (64). After surgical separation of the parabiosed adult and old mice, the adult LN returned to normal, while the old remained hypocellular. While joined, the frequencies and numbers of stromal subsets in the adult parabiont were similar to the old parabiont than to those in isochronic parabiosis (adult–adult pairs). Together, these results suggest that a circulating soluble or cellular factor, present in the old parabiont, can influence the structure and cellularity of the adult LN *in trans*. Research is in progress to test this hypothesis.

### Possible Molecular Targets to Rejuvenate Aging LN

Based on the observed defects, discussed above, one can hypothesize about candidate molecular pathways responsible for LN defects. One attractive target is LT signaling. The receptor for LTβ (LTβR) is expressed on LECs, HEVs, FRCs, and FDCs (28, 39). The bidirectional relationship between T cells and FRCs in homeostasis is critical for both populations of cells (23). Known producers of LT, such as DCs, B cells, and T cells (37) are less abundant in the aged LN (15, 49). When LTβ is conditionally depleted in young mice, there is a decline in LN organization and impaired induction of antiviral immune responses (37), similar to that described in aged LN (14, 15).

Approaches that limit fibrosis should also be considered. Regardless of tissue type, aging is the biggest risk factor for fibrosis (52), including in skin-draining LN in humans (34, 35). Serum levels of transforming growth factor-beta (TGF-β) are increased with aging in both mice and humans (65) and may be related to the age-associated increases in T-regulatory cells (10, 53, 66). Within the LN, fibrosis has been most studied in the context of simian immunodeficiency virus (SIV) in non-human primates (55). Increased levels of TGF-β, pSMAD2,3 signaling, and increased levels of fibrosis were found in LN of SIV-infected animals, where immune reconstitution is limited after antiretroviral therapy (55). As SIV infection proceeds, naïve T cells in fibrotic regions undergo apoptosis (10, 67). Administration of the anti-fibrotic drug pirfenidone to reverse fibrosis restored naïve CD4^+^ T cell populations in SIV-infected monkeys in combination with antiretroviral therapy (55). Along these lines, we have observed increased fibrosis in aged mouse LN compared to adults. Understanding the interactions between FRCs and lymphocytes, and how fibrosis may impact these communications could have important therapeutic potential (10).

## Conclusion

Age-related changes to LN stroma are emerging as an important area of research. Full understanding of these changes will likely be critical to understand, and, perhaps, correct age-related disorganization of T cell homeostasis and immune function. LN stroma is critical for naïve T cell homeostasis, providing both chemokine gradients for effective trafficking into the LN, and survival signals to the naïve T cell upon arrival (7, 13). Further, stromal cells control influx of antigen, and there is initial evidence that this process may be adversely affected by aging LN (15, 16). Within the LN, FRCs (13) and FDCs (14), both decline numerically and exhibit disorganized network formation, with a potential to impair interactions with T and B cells. A key challenge in front of us is to (1) understand how aging alters the structure and function of each of the stromal cellular components and their interaction and (2) dissect the functional consequences of such changes for protective immunity. The ultimate goal should be to manipulate and restore stromal cell function in response to vaccination or infection and thus provide new targets to improve immunity in the elderly.

## Author Contributions

HT, MS, and JN-Z wrote the paper. CS contributed to critically revising the paper. All the authors extensively discussed the topic, read and approved the final version of the manuscript.

## Conflict of Interest Statement

The authors declare no conflicts of interest. JN-Z is an unpaid Scientific Advisory Board member of Organic Vaccines, Inc., an entity that had no input into any part of studies reported here.

## Abbreviations

BEC, blood endothelial cells; DC, dendritic cell; ECM, extracellular matrix; FDC, follicular dendritic cell; FRC, fibroblastic reticular cell; HEV, high endothelial venule; ILC, innate lymphoid cells; LEC, lymphatic endothelial cell; LN, lymph node; LTi, lymphoid tissue inducer; LTo, lymphoid tissue organizer; SCS, subcapsulary sinus.

## References

[1] Briceño O Lissina A Wanke K Afonso G Braun von A Ragon K Reduced naïve CD8(+) T-cell priming efficacy in elderly adults. Aging Cell (2016) 15:14–21. 10.1111/acel.12384 26472076 PMC4717282

[2] Nikolich-Žugich J. Aging of the T cell compartment in mice and humans: from no naive expectations to foggy memories. J Immunol (2014) 193:2622–9. 10.4049/jimmunol.1401174 25193936 PMC4157314

[3] Nikolich-Žugich J Davies JS Homeostatic migration and distribution of innate immune cells in primary and secondary lymphoid organs with aging. Clin Exp Immunol (2016) 187:337–44. 10.111./cei.12920 PMC5290228 28035684

[4] Nikolich-Žugich J Li G Uhrlaub JL Renkema KR Smithey MJ. Age-related changes in CD8 T cell homeostasis and immunity to infection. Semin Immunol (2012) 24:356–64. 10.1016/j.smim.2012.04.009 22554418 PMC3480557

[5] Chinn IK Blackburn CC Manley NR Sempowski GD. Changes in primary lymphoid organs with aging. Semin Immunol (2012) 24:309–20. 10.1016/j.smim.2012.04.005 22559987 PMC3415579

[6] Wertheimer AM Bennett MS Park B Uhrlaub JL Martinez C Pulko V Aging and cytomegalovirus infection differentially and jointly affect distinct circulating T cell subsets in humans. J Immunol (2014) 192:2143–55. 10.4049/jimmunol.1301721 24501199 PMC3989163

[7] Link A Vogt TK Favre S Britschgi MR Acha-Orbea H Hinz B Fibroblastic reticular cells in lymph nodes regulate the homeostasis of naive T cells. Nat Immunol (2007) 8:1255–65. 10.1038/ni1513 17893676

[8] Bajénoff M Egen JG Qi H Huang AYC Castellino F Germain RN. Highways, byways and breadcrumbs: directing lymphocyte traffic in the lymph node. Trends Immunol (2007) 28:346–52. 10.1016/j.it.2007.06.005 17625969

[9] Bajénoff M Germain RN. B-cell follicle development remodels the conduit system and allows soluble antigen delivery to follicular dendritic cells. Blood (2009) 114:4989–97. 10.1182/blood-2009-06-229567 19713459 PMC2788973

[10] Fletcher AL Acton SE Knoblich K. Lymph node fibroblastic reticular cells in health and disease. Nat Rev Immunol (2015) 15:350–61. 10.1038/nri3846 25998961 PMC5152733

[11] Fletcher AL Malhotra D Acton SE Lukacs-Kornek V Bellemare-Pelletier A Curry M Reproducible isolation of lymph node stromal cells reveals site-dependent differences in fibroblastic reticular cells. Front Immunol (2011) 2:35. 10.3389/fimmu.2011.00035 22566825 PMC3342056

[12] Benezech C Nayar S Finney BA Withers DR Lowe K Desanti GE CLEC-2 is required for development and maintenance of lymph nodes. Blood (2014) 123:3200–7. 10.1182/blood-2013-03-489286 24532804 PMC4023425

[13] Becklund BR Purton JF Ramsey C Favre S Vogt TK Martin CE The aged lymphoid tissue environment fails to support naïve T cell homeostasis. Sci Rep (2016) 6:30842. 10.1038/srep30842 27480406 PMC4969611

[14] Turner VM Mabbott NA. Structural and functional changes to lymph nodes in ageing mice. Immunology (2017) 151(2):239–47. 10.1111/imm.12727 28207940 PMC5418465

[15] Richner JM Gmyrek GB Govero J Tu Y van der Windt GJW Metcalf TU Age-dependent cell trafficking defects in draining lymph nodes impair adaptive immunity and control of *West Nile virus* infection. PLoS Pathog (2015) 11:e1005027. 10.1371/journal.ppat.1005027 26204259 PMC4512688

[16] Zolla V Nizamutdinova IT Scharf B Clement CC Maejima D Akl T Aging-related anatomical and biochemical changes in lymphatic collectors impair lymph transport, fluid homeostasis, and pathogen clearance. Aging Cell (2015) 14:582–94. 10.1111/acel.12330 25982749 PMC4531072

[17] Aebischer D Iolyeva M Halin C The inflammatory response of lymphatic endothelium. Angiogenesis (2013) 17:383–93. 10.1007/s10456-013-9404-3 24154862

[18] Girard J-P Moussion C Förster R HEVs, lymphatics and homeostatic immune cell trafficking in lymph nodes. Nat Rev Immunol (2012) 12:762–73. 10.1038/nri3298 23018291

[19] Bajénoff M Egen JG Koo LY Laugier JP Brau F Glaichenhaus N Stromal cell networks regulate lymphocyte entry, migration, and territoriality in lymph nodes. Immunity (2006) 25:989–1001. 10.1016/j.immuni.2006.10.011 17112751 PMC2692293

[20] Malhotra D Astarita J Lukacs-Kornek V Tayalia P Gonzalez SF Chang SK Transcriptional profiling of stroma from inflamed and resting lymph nodes defines immunological hallmarks. Nat Immunol (2012) 13:499–510. 10.1038/ni.2262 22466668 PMC3366863

[21] Heesters BA Myers RC Carroll MC. Follicular dendritic cells: dynamic antigen libraries. Nat Rev Immunol (2014) 14:495–504. 10.1038/nri3689 24948364

[22] Sá da Bandeira D Casamitjana J Crisan M. Pericytes, integral components of adult hematopoietic stem cell niches. Pharmacol Ther (2017) 171:104–13. 10.1016/j.pharmthera.2016.11.006 27908803

[23] Katakai T Hara T Sugai M Gonda H Shimizu A. Lymph node fibroblastic reticular cells construct the stromal reticulum via contact with lymphocytes. J Exp Med (2004) 200:783–95. 10.1084/jem.20040254 15381731 PMC2211971

[24] Bar-Ephraïm YE Mebius RE Innate lymphoid cells in secondary lymphoid organs. Immunol Rev (2016) 271:185–99. 10.1111/imr.12407 27088915

[25] Artis D Spits H. The biology of innate lymphoid cells. Nature (2015) 517:293–301. 10.1038/nature14189 25592534

[26] Cupedo T Kraal G Mebius RE The role of CD45+CD4+CD3– cells in lymphoid organ development. Immunol Rev (2002) 189:41–50. 10.1034/j.1600-065X.2002.18905.x 12445264

[27] Scandella E Bolinger B Lattmann E Miller S Favre S Littman DR Restoration of lymphoid organ integrity through the interaction of lymphoid tissue-inducer cells with stroma of the T cell zone. Nat Immunol (2008) 9:667–75. 10.1038/ni.1605 18425132

[28] Zhu M Fu Y-X. The role of core TNF/LIGHT family members in lymph node homeostasis and remodeling. Immunol Rev (2011) 244:75–84. 10.1111/j.1600-065X.2011.01061.x 22017432

[29] Acton SE Astarita JL Malhotra D Lukacs-Kornek V Franz B Hess PR Podoplanin-rich stromal networks induce dendritic cell motility via activation of the C-type lectin receptor CLEC-2. Immunity (2012) 37:276–89. 10.1016/j.immuni.2012.05.022 22884313 PMC3556784

[30] Acton SE Farrugia AJ Astarita JL Mourão-Sá D Jenkins RP Nye E Dendritic cells control fibroblastic reticular network tension and lymph node expansion. Nature (2014) 514:498–502. 10.1038/nature13814 25341788 PMC4235005

[31] Astarita JL Cremasco V Fu J Darnell MC Peck JR Nieves-Bonilla JM The CLEC-2-podoplanin axis controls the contractility of fibroblastic reticular cells and lymph node microarchitecture. Nat Immunol (2015) 16:75–84. 10.1038/ni.3035 25347465 PMC4270928

[32] Masters AR Haynes L Su DM Palmer DB Immune senescence: significance of the stromal microenvironment. Clin Exp Immunol (2016) 187:6–15. 10.1111/cei.12851 27529161 PMC5167042

[33] Luscieti P Hubschmid T Cottier H Hess MW Sobin LH. Human lymph node morphology as a function of age and site. J Clin Pathol (1980) 33:454–61. 10.1136/jcp.33.5.454 7400343 PMC1146110

[34] Denz FA Age changes in lymph nodes. J Pathol Bacteriol (1947) 59:575–91. 10.1002/path.1700590409 18907567

[35] Maiborodin IV Agzaev MK Ragimova TM Maiborodin II Age-related changes in the structure of lymphoid organs: a review of the literature. Adv Gerontol (2016) 6:282–90. 10.1134/S2079057016040081 28514539

[36] Aw D Hilliard L Nishikawa Y Cadman ET Lawrence RA Palmer DB. Disorganization of the splenic microanatomy in ageing mice. Immunology (2016) 148:92–101. 10.1111/imm.12590 26840375 PMC4819137

[37] Junt T Scandella E Ludewig B. Form follows function: lymphoid tissue microarchitecture in antimicrobial immune defence. Nat Rev Immunol (2008) 8:764–75. 10.1038/nri2414 18825130

[38] Heesters BA Chatterjee P Kim Y-A Gonzalez SF Kuligowski MP Kirchhausen T Endocytosis and recycling of immune complexes by follicular dendritic cells enhances B cell antigen binding and activation. Immunity (2013) 38:1164–75. 10.1016/j.immuni.2013.02.023 23770227 PMC3773956

[39] Onder L Danuser R Scandella E Firner S Chai Q Hehlgans T Endothelial cell-specific lymphotoxin-β receptor signaling is critical for lymph node and high endothelial venule formation. J Exp Med (2013) 210:465–73. 10.1084/jem.20121462 23420877 PMC3600902

[40] Donato AJ Morgan RG Walker AE Lesniewski LA. Cellular and molecular biology of aging endothelial cells. J Mol Cell Cardiol (2015) 89:122–35. 10.1016/j.yjmcc.2015.01.021 25655936 PMC4522407

[41] Katakai T Hara T Lee J-H Gonda H Sugai M Shimizu A. A novel reticular stromal structure in lymph node cortex: an immuno-platform for interactions among dendritic cells, T cells and B cells. Int Immunol (2004) 16:1133–42. 10.1093/intimm/dxh113 15237106

[42] Malhotra D Fletcher AL Turley SJ. Stromal and hematopoietic cells in secondary lymphoid organs: partners in immunity. Immunol Rev (2013) 251:160–76. 10.1111/imr.12023 23278748 PMC3539229

[43] Förster R Davalos-Misslitz AC Rot A. CCR7 and its ligands: balancing immunity and tolerance. Nat Rev Immunol (2008) 8:362–71. 10.1038/nri2297 18379575

[44] Tan JT Dudl E LeRoy E Murray R Sprent J Weinberg KI IL-7 is critical for homeostatic proliferation and survival of naive T cells. Proc Natl Acad Sci U S A (2001) 98:8732–7. 10.1073/pnas.161126098 11447288 PMC37504

[45] Surh CD Sprent J Homeostasis of naive and memory T cells. Immunity (2008) 29:848–62. 10.1016/j.immuni.2008.11.002 19100699

[46] Cremasco V Woodruff MC Onder L Cupovic J Nieves-Bonilla JM Schildberg FA B cell homeostasis and follicle confines are governed by fibroblastic reticular cells. Nat Immunol (2014) 15:973–81. 10.1038/ni.2965 25151489 PMC4205585

[47] den Braber I Mugwagwa T Vrisekoop N Westera L Mögling R de Boer AB Maintenance of peripheral naive T cells is sustained by thymus output in mice but not humans. Immunity (2012) 36:288–97. 10.1016/j.immuni.2012.02.006 22365666

[48] Textor J Mandl JN de Boer RJ. The reticular cell network: a robust backbone for immune responses. PLoS Biol (2016) 14:e2000827. 10.1371/journal.pbio.2000827 27727272 PMC5058469

[49] Uhrlaub JL Pulko V DeFilippis VR Broeckel R Streblow DN Coleman GD Dysregulated TGF-β production underlies the age-related vulnerability to *Chikungunya virus*. PLoS Pathog (2016) 12:e1005891. 10.1371/journal.ppat.1005891 27736984 PMC5063327

[50] Chang JE Turley SJ. Stromal infrastructure of the lymph node and coordination of immunity. Trends Immunol (2015) 36:30–9. 10.1016/j.it.2014.11.003 25499856

[51] Shoulders MD Raines RT. Collagen structure and stability. Annu Rev Biochem (2009) 78:929–58. 10.1146/annurev.biochem.77.032207.120833 19344236 PMC2846778

[52] Thannickal VJ Zhou Y Gaggar A Duncan SR. Fibrosis: ultimate and proximate causes. J Clin Invest (2014) 124:4673–7. 10.1172/JCI74368 25365073 PMC4347226

[53] Borthwick LA Wynn TA IL-13 and TGF-β1: core mediators of fibrosis. Curr Pathobiol Rep (2015) 3:273–82. 10.1007/s40139-015-0091-1

[54] Ahmadi O McCall JL Stringer MD. Does senescence affect lymph node number and morphology? A systematic review. ANZ J Surg (2013) 83:612–8. 10.1111/ans.12067 23347421

[55] Estes JD Reilly C Trubey CM Fletcher CV Cory TJ Michael Piatak J Antifibrotic therapy in simian immunodeficiency virus infection preserves CD4+ T-cell populations and improves immune reconstitution with antiretroviral therapy. J Infect Dis (2015) 211:744–54. 10.1093/infdis/jiu519 25246534 PMC4334805

[56] Zeng M Smith AJ Wietgrefe SW Southern PJ Schacker TW Reilly CS Cumulative mechanisms of lymphoid tissue fibrosis and T cell depletion in HIV-1 and SIV infections. J Clin Invest (2011) 121:998–1008. 10.1172/JCI45157 21393864 PMC3049394

[57] Jarjour M Jorquera A Mondor I Wienert S Narang P Coles MC Fate mapping reveals origin and dynamics of lymph node follicular dendritic cells. J Exp Med (2014) 211:1109–22. 10.1084/jem.20132409 24863064 PMC4042641

[58] Kranich J Krautler NJ. How follicular dendritic cells shape the B-cell antigenome. Front Immunol (2016) 7:225. 10.3389/fimmu.2016.00225 27446069 PMC4914831

[59] Chen LL Adams JC Steinman RM Anatomy of germinal centers in mouse spleen, with special reference to “follicular dendritic cells.”. J Cell Biol (1978) 77:148–64. 10.1083/jcb.77.1.148 659510 PMC2110033

[60] Pinti M Appay V Campisi J Frasca D Fülöp T Sauce D Aging of the immune system – focus on inflammation and vaccination. Eur J Immunol (2016) 46:2286–301. 10.1002/eji.201546178 27595500 PMC5156481

[61] Conboy MJ Conboy IM Rando TA. Heterochronic parabiosis: historical perspective and methodological considerations for studies of aging and longevity. Aging Cell (2013) 12:525–30. 10.1111/acel.12065 23489470 PMC4072458

[62] Smith LK He Y Park J-S Bieri G Snethlage CE Lin K β2-microglobulin is a systemic pro-aging factor that impairs cognitive function and neurogenesis. Nat Med (2015) 21:932–7. 10.1038/nm.3898 26147761 PMC4529371

[63] Villeda SA Plambeck KE Middeldorp J Castellano JM Mosher KI Luo J Young blood reverses age-related impairments in cognitive function and synaptic plasticity in mice. Nat Med (2014) 20:659–63. 10.1038/nm.3569 24793238 PMC4224436

[64] Davies JS Thompson HL Pulko V Padilla-Torres J Nikolich-Zugich J Role of cell-intrinsic and environmental age-related changes in altered maintenance of murine T cells in lymphoid organs. J Gerontol A Biol Sci Med Sci (2017):glx102 10.1093/gerona/glx102 PMC6037132 28582491

[65] Carlson ME Conboy MJ Hsu M Barchas L Jeong J Agrawal A Relative roles of TGF-β1 and Wnt in the systemic regulation and aging of satellite cell responses. Aging Cell (2009) 8:676–89. 10.1111/j.1474-9726.2009.00517.x 19732043 PMC2783265

[66] Garg SK Delaney C Toubai T Ghosh A Reddy P Banerjee R Aging is associated with increased regulatory T-cell function. Aging Cell (2014) 13:441–8. 10.1111/acel.12191 24325345 PMC4032602

[67] Zeng M Paiardini M Engram JC Beilman GJ Chipman JG Schacker TW Critical role of CD4 T cells in maintaining lymphoid tissue structure for immune cell homeostasis and reconstitution. Blood (2012) 120:1856–67. 10.1182/blood-2012-03-4186 22613799 PMC3433090

